# Response on “Commentary on “Is gaitrite system sensitive in discriminating gait pattern of subjects affected by Charcot Marie tooth? A pilot study””

**DOI:** 10.1007/s10072-026-08940-6

**Published:** 2026-03-09

**Authors:** Cristina Schenone, Marta Ponzano, Laura Mori

**Affiliations:** 1 Department of Neuroscience, Rehabilitation, Ophthalmology, Genetics, Maternal and Child Health (DINOGMI), University of Genoa, Genoa, Italy; 2 Azienda Ospedaliera Metropolitana Regione Liguria (AOM) Plesso Ospedale Policlinico San Martino IRCCS, Genoa, Italy; 3 Department of Health Sciences (DISSAL), Biostatistics Unit, University of Genoa, Genoa, Italy

Dear Editor-in-chief,

We sincerely thank Dr. Gouelle for his constructive comments, which provide valuable insights into important methodological aspects of instrumental gait analysis. We appreciate the opportunity to further clarify the scope and the exploratory nature of our work.

As specified in the manuscript, our study was designed as a pilot, cross-sectional investigation, with the primary aim of exploring the feasibility and potential utility of an instrumented walkway system in patients with Charcot–Marie–Tooth disease (CMT). Our conclusions were intentionally cautious, avoiding any claim regarding disease severity stratification or longitudinal sensitivity to progression.

As we stated in the paper, GAITRite system has already been recognized to be sensitive in discriminating healthy controls from patients in many neurological diseases [1–4].

Our aim was not to provide a comprehensive characterization of gait domains, variability components, or disease progression, but rather to offer preliminary data supporting the use of a quick and clinically accessible instrumental assessment in CMT patients. In this context, the term “sensitive” in the title was used in a restricted sense, referring to the ability of the system to distinguish between CMT patients and healthy controls in our sample.

We share the view that considerations regarding reliability indices and minimum detectable change are essential. Since our study was cross-sectional and not aimed at assessing within-subject change over time, these aspects were beyond its scope.

Regarding statistical analysis, as an exploratory pilot study, we acknowledge some limitations that could be addressed in future research. In addition to the small sample size, a further limitation is that our analysis was restricted to a set of bivariate comparisons. In our study, gait speed was not treated as a covariate because reduced walking speed represents a central feature of functional impairment in CMT and reflects part of the clinical phenotype captured by both clinical scales and instrumental measures. We hope that these initial findings will serve as a foundation for future studies that more comprehensively address potential confounding factors such as walking speed.

Finally, we appreciate the opportunity to clarify the distinction between GAITRite hardware and PKMAS software, as we described in the Methods section. We agree that transparency regarding acquisition and processing algorithms is fundamental in instrumental gait research.

In conclusion, we view Dr. Gouelle’s remarks as a constructive contribution that highlights important methodological standards for future studies. We hope that our findings will offer preliminary evidence supporting the feasibility and potential clinical utility of instrumented gait analysis in CMT, to be confirmed in larger and methodologically more comprehensive studies.
